# Error-related activity of the sensorimotor network contributes to the prediction of response to cognitive-behavioral therapy in obsessive–compulsive disorder

**DOI:** 10.1016/j.nicl.2022.103216

**Published:** 2022-09-30

**Authors:** Rosa Grützmann, Julia Klawohn, Björn Elsner, Benedikt Reuter, Christian Kaufmann, Anja Riesel, Katharina Bey, Stephan Heinzel, Norbert Kathmann

**Affiliations:** a Humboldt-Universität zu Berlin, Department of Psychology, Germany; b MSB Medical School Berlin, Department of Psychology, Germany; c MSB Medical School Berlin, Department of Medicine, Germany; d Universität Hamburg, Department of Psychology, Germany; e University Hospital Bonn, Department of Psychiatry and Psychotherapy, Germany; f Freie Universität Berlin, Department of Education and Psychology, Germany

**Keywords:** Obsessive–compulsive disorder, Error monitoring, Cognitive-behavioral therapy, Response prediction, FMRI, OCD, Obsessive-compulsive disorder, CBT, Cognitive-behavioral therapy

## Abstract

**Background:**

Although cognitive behavioral therapy is a highly effective treatment for obsessive–compulsive disorder (OCD), yielding large symptom reductions on the group level, individual treatment response varies considerably. Identification of treatment response predictors may provide important information for maximizing individual treatment response and thus achieving efficient treatment resource allocation. Here, we investigated the predictive value of previously identified biomarkers of OCD, namely the error-related activity of the supplementary motor area (SMA) and the sensorimotor network (SMN, postcentral gyrus/precuneus).

**Methods:**

Seventy-two participants with a primary diagnosis of OCD underwent functional magnetic resonance imaging (fMRI) scanning while performing a flanker task prior to receiving routine-care CBT.

**Results:**

Error-related BOLD response of the SMN significantly contributed to the prediction of treatment response beyond the variance accounted for by clinical and sociodemographic variables. Stronger error-related SMN activity at baseline was associated with a higher likelihood of treatment response.

**Conclusions:**

The present results illustrate that the inclusion of error-related SMN activity can significantly increase treatment response prediction quality in OCD. Stronger error-related activity of the SMN may reflect the ability to activate symptom-relevant processing networks and may thus facilitate response to exposure-based CBT interventions.

## Highlights

• Error-related brain activity contributes to therapy response prediction in OCD.
• Higher activity of the sensorimotor network predicts higher likelihood of response.
• Sensorimotor network activity may contribute to “not-just-right”-experiences.

## 1 Introduction

Obsessive-compulsive disorder (OCD) is characterized by repetitive and distressing obsessions and compulsions (American Psychiatric Association, 2013). OCD can lead to a significant impairment in everyday functioning and quality of life and often follows a chronic course when not treated (Koran, Thienemann, & Davenport, 1996).

Cognitive-behavioral therapy (CBT) is a highly effective treatment for OCD yielding very large effects on primary outcome measures compared to wait list or placebo in randomized controlled trials (*d* ∼ 1.30, Olatunji et al., 2013, Öst et al., 2015) and under routine care conditions (*d* = 1.47, Kathmann, Jacobi, Elsner, & Reuter, 2022). However, individual treatment responses vary considerably. While about 65 % of the treated patients show clinically significant symptom change and about 50 % reach remission, about 25 % to 30 % of the patients who begin CBT discontinue the treatment prematurely (Abramowitz, 2006, Kathmann et al., 2022, Öst et al., 2015, Simpson et al., 2006).

Personalized medicine aims to identify individual clinical, sociodemographic, behavioral and neurobiological features that are associated with reduced treatment response in order to subsequently use this information for individualized treatment allocation or optimization (Ozomaro, Wahlestedt, & Nemeroff, 2013). An approach that promises high efficiency and practicability is using clinical and demographical data that are routinely acquired in most treatment settings. Among these variables, clinical (initial symptom severity, comorbid personality disorders, hoarding subtype) and sociodemographic characteristics (unemployment, family dysfunction, relationship status) and previous treatment experience (previous medication use) were identified as predictors (Kathmann et al., 2022, Keeley et al., 2008, Knopp et al., 2013). While comorbid personality disorders, hoarding subtype, unemployment, higher family dysfunction and being single/unmarried were associated with worse therapy response, higher initial symptom severity and previous medication use predicted better therapy response. As multiple predictors may contribute and interact simultaneously, data-driven approaches have also been applied to more closely capture these dynamics (Hilbert et al., 2021, Hilbert et al., 2020). However, machine learning modeling of treatment response in OCD based on routine clinical and sociodemographic data did not substantially outperform the classification accuracy of regression analyses (Hilbert et al., 2021), thus implying possible limitations of this data type.

The inclusion of additional data sources, such as (neuro)biological data, may further improve therapy outcome prediction (Paul et al., 2022). Biomarkers may provide higher objectivity than self-report or interview data and may more closely capture underlying pathogenic mechanisms (Gottesman and Gould, 2003, Riesel et al., 2021). Thus, although they are more difficult to assess than sociodemographic or clinical data, biomarkers pose a promising candidate for therapy outcome predictors.

Previous research indicates that excessive error signals may present a biomarker or endophenotype for OCD. Increased error-related activity brain activity in OCD compared to healthy control participants (HC) is commonly observed in the cingulo-opercular salience network, comprising the dorsal anterior cingulate cortex, SMA, insula/ frontal operculum and rostral anterior cingulate cortex (Grutzmann et al., 2016, Grützmann et al., 2021, Norman et al., 2019). Additionally, increased error-related activity has also been observed in the default mode network (DMN), the sensorimotor network (SMN) and the fronto-limbic emotion processing network in OCD (Fitzgerald et al., 2010, Grützmann et al., 2021, Stern et al., 2011). The hyperactivation of the salience network is also captured in the error-related negativity (ERN), an event-related potential component in the EEG, which is robustly increased in OCD patients (Riesel, 2019). Overactive performance monitoring as reflected in these excessive error signals may trigger the frequent “not-just-right” feelings and intrusive harm-related thoughts in OCD patients and thus contribute to the repetitive compulsive loops (Pitman, 1987). As increased error signals are not only observed in OCD, but also in anxiety disorders (Endrass and Ullsperger, 2014, Riesel et al., 2017, Weinberg et al., 2015), and in unaffected first-degree relatives of patients with OCD or anxiety disorders (Riesel et al., 2011, Riesel et al., 2019), they might represent an overarching risk marker for internalizing disorders, possibly reflecting underlying trait-like threat sensitivity (Weinberg et al., 2016). Taken together, increased error-related brain activity might reflect a central pathogenic mechanism in OCD and thus a promising candidate for a treatment response predictor.

Neuroimaging studies show that indicators of resting state functional connectivity and task-related brain activity can serve as predictors of CBT treatment response in OCD. Parameters reflecting altered resting-state functional connectivity of the DMN and visual network (Feusner et al., 2015, Reggente et al., 2018), predicted better response beyond the contribution of clinical, sociodemographic and behavioral data (i.e. Stroop interference). Fullana et al. (2017) showed that a lower resting-state functional connectivity between the basolateral amygdala and the ventromedial prefrontal cortex, that might be functionally associated with reduced capacity to regulate fear responses, also positively predicted treatment response. Similar effects have been observed for task-related activity. Olatunji et al. (2014) showed in a small sample of OCD patients with contamination/cleaning symptoms that higher activation of the emotion-processing network (i.e. anterior temporal pole, amygdala) and the SMN (i.e. postcentral gyrus, precuneus) during symptom provocation were associated with a stronger treatment response. Furthermore, two recent studies showed that stronger conflict-related activity of the salience network was associated with an increased treatment response (Norman et al., 2021, Pagliaccio et al., 2019). Taken together, these studies imply that patients with stronger baseline abnormalities in brain activity are more likely to improve during CBT. On a functional level, stronger brain activity abnormalities at baseline may provide a larger potential to achieve a more adaptive brain state through therapeutic interventions.

Previous research indicates that excessive error-related activity may play an important role in OCD pathology. Specifically, it might functionally contribute to characteristic features of OCD symptomatology such as increased self-monitoring, error aversiveness, perfectionism, and rigidity/over-controlled behavior (Norman et al., 2019, Pitman, 1987, Riesel, 2019). Despite this potential as a functional biomarker, to the best of our knowledge error-related activity of has not been tested as a therapy outcome predictor in OCD. The present analysis uses data from OCD patients who were on a waitlist for CBT treatment at the outpatient clinic of the Humboldt-Universität zu Berlin and had received fMRI scanning while performing a flanker task (Grützmann et al., 2021). Analysis of the fMRI data identified increased error-related BOLD response of regions within the salience network (SMA) and SMN^1^ (precuneus, postcentral gyrus) in the patient group. Patients then received routine-care non-manualized CBT accompanied by standardized diagnostic assessments. Here, we investigate whether inclusion of error-related activity of the salience network and SMN can improve treatment response prediction in patients with OCD beyond the contribution of previously established clinical and sociodemographic predictors. In accordance with previous studies, we expected a higher likelihood of a treatment response in patients with stronger error-related activation.

## 2 Method and materials

### 2.1 Participants

**Fig. 1:**
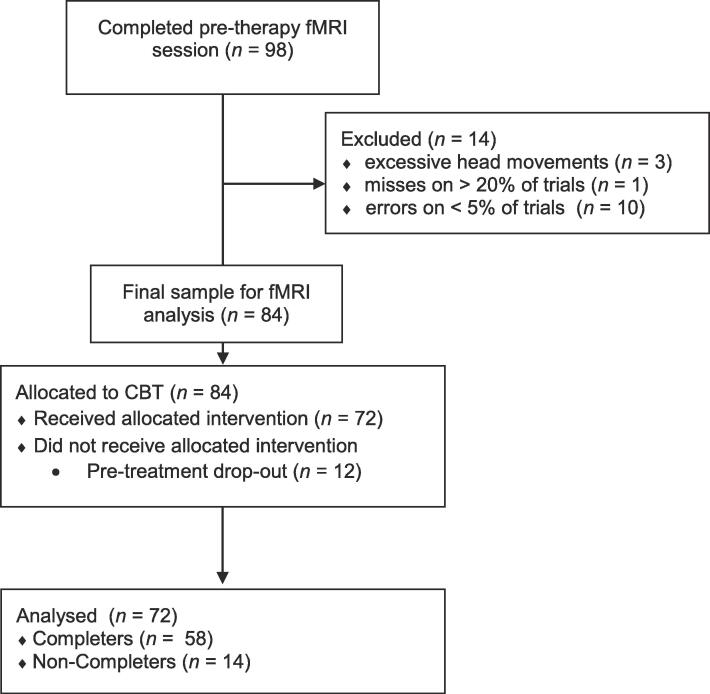
Participant flow from the pre-therapy fMRI session to the final data analysis. FMRI = functional magnetic resonance imaging, CBT = cognitive-behavioral therapy.

Participant flow is illustrated in Fig. 1. Initially, 98 patients with OCD took part in the pre-therapy fMRI data collection. Patients had a primary diagnosis of OCD, as assessed by trained clinicians using the German version of the Structured Clinical Interview for DSM-IV (First, Spitzer, Gibbon, & Williams, 1995), with a severity score of > 12 in the Yale-Brown Obsessive Compulsive Scale (Y-BOCS; Goodman et al., 1989). Exclusion criteria were prominent suicidal ideation, any lifetime substance dependence, borderline personality disorder, comorbid psychotic disorders, history of head trauma and neurological diseases. All participants received verbal and written explanation of the purpose and procedures of the study, gave their written informed consent in accordance with the ethical guidelines of the Declaration of Helsinki. They received 10 € per hour for the participation in the fMRI experiment. Fourteen patients had to be excluded from the fMRI data analysis due to poor data quality (*n* = 3) or failure to comply with experimental instructions (*n* = 11), resulting in a final analysis sample of 84 patients. For the treatment response prediction, all patients that received at least one therapy session were included (*n* = 72). Fourteen of these patients prematurely discontinued treatment (non-completers), as defined by the patient’s unilateral decision to abandon treatment without the therapist’s approval, while 58 patients completed the treatment (completers), as defined by a consensual termination decision of patient and therapist based on clinical criteria. Patients were treated with CBT including exposure and response prevention and cognitive therapy (Foa, Yadin, & Lichner, 2012) delivered by licensed, experienced therapists with at least three years of training in CBT, who participated in weekly intervision sessions. Treatments were conducted in accordance with the general regulations for psychotherapy in the public German health care system, which allows up to 80 units of 50 min each per treatment. For patients in the present study, the mean number of therapy sessions was 46 (SD: 19.28, range: 8–80). Sessions usually took place once or twice weekly, but therapists were free to adjust session length when implementing exposure and to reduce session frequency at the end of treatment. The mean therapy duration was 74 weeks (SD: 34, range: 19–150). The mean therapy duration was significantly shorter in the non-completers (M: 50, SD: 20) than in the completers (M: 80, SD: 34), *t*(70) = 4.36, *p* <.001. Mean frequency of therapy sessions was 0.67 (SD: 0.27, range: 0.25 – 1.90), indicating that on average patients received less than one therapy session each week. Please note, that this value very likely underestimates the true session frequency in active therapy periods, as the total therapy duration also contains periods in which the outpatient therapy was paused, for example due to vacation. The mean frequency of therapy sessions did not significantly differ between completers (M: 0.68, SD: 0.28) and non-completers (M: 0.63, SD: 0.19), *t*(70) = 0.73, *p* =.470.

Forty-seven patients (65 %) showed at least one comorbid disorder including affective disorders (depressive episode *n* = 20, recurrent depressive disorder *n* = 16, dysthymic disorder *n* = 7), anxiety disorders (agoraphobia *n* = 2, social phobia *n* = 5, specific phobia *n* = 7, panic disorder *n* = 1, generalized anxiety disorder *n* = 3), somatoform disorders (undifferentiated somatoform disorder *n* = 2, hypochondriacal disorder *n* = 1, other somatoform disorders n = 1), post-traumatic stress disorder (*n* = 1) and eating disorders (atypical anorexia nervosa *n* = 1). At the pre-therapy fMRI measurement, 33 patients (45 %) reported taking one or more psychotropic medications in the last three months (SSRI, *n* = 27; SSNRI, *n* = 4; tricyclic antidepressants, *n* = 4; tetracyclic antidepressant, *n* = 1). Further characteristics of the sample at pre-therapy baseline are presented in Table 1.

**Table 1:** Socio-demographic and clinical characteristics of the whole sample (all), completers and non-completers at baseline (pre-therapy).

	All		Completer		Non-Completer	
Sex male/female	31/41		26/32		5/9	
Unemployed *n* (%)	12 (16 %)		12 (21 %)		0 (0 %)	
Any comorbid axis-I disorder *n* (%)	47 (65 %)		38 (65 %)		9 (64 %)	
Any comorbid personality disorder *n* (%)	13 (17 %)		11 (19 %)		2 (14 %)	
Any psychotropic medication *n* (%)	33 (45 %)		26 (44 %)		7 (50 %)	
	*M*	*SD*	*M*	*SD*	*M*	*SD*
Age	31.47	8.94	32.60	9.04	26.79	6.97
Number of therapy sessions	46.24	19.28	50.26	18.47	29.57	12.75

## 3 Data collection

### 3.1 FMRI data

**Fig. 2:**
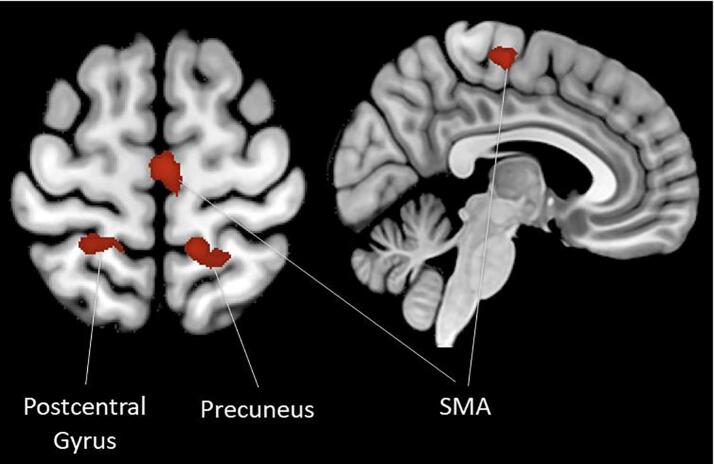
Brain regions with increased error-related activity in OCD patients compared to healthy control participants (Grützmann et al., 2021). Beta values from these clusters were extracted and served as predictors in the regression analyses.

The experimental design, data analysis and results of the pre-therapy fMRI experiment are described in detail in Grützmann et al. (2021). Briefly, participants performed an arrow-version of the flanker task (Eriksen and Eriksen, 1974, Kopp et al., 1996) while error-related BOLD response was assessed with a 3-Tesla Siemens Trio MR system. Error- and conflict related brain activity was assessed in OCD patients, unaffected first-degree relatives of OCD patients and healthy control participants. Exclusion criteria for the control group were psychoactive medication in the past three months, any current or past axis-I psychological disorder, and family history of OCD in first-degree relatives. After standard preprocessing with SPM12 (Statistical Parametric Mapping Version 7487, https://www.fil.ion.ucl.ac.uk/spm) comprising realignment, movement and slice time correction and normalization to the standard template provided by the Montreal Neurological Institute, the data were analyzed with general linear model specifying two regressors of interest assessing error- (by modeling incongruent error > incongruent correct) and conflict-related activity (by modeling incongruent correct > congruent correct). Error-related BOLD response was then compared between the patient group and a healthy control group, consisting of 99 participants matched for gender, age, education, and handedness. To correct for multiple comparisons, an extent threshold correction as defined by Monte Carlo simulations (3DClustSim; implemented in AFNI; Cox, 1996) was applied. For a threshold at the voxel level of *p* <.001 uncorrected, and spatial properties of the current study, 10.000 simulations resulted in an extent threshold of 56 voxels at *p* <.05. The analysis identified increased error-related activity in three brain regions in OCD patients, namely the SMA, the postcentral gyrus (PCG) and the precuneus (see Fig. 2 and Table 2). Beta values from these clusters (defined as 5 mm sphere radius around the MNI coordinates of peak voxel in the whole brain interaction) were extracted and served as predictors in the regression analyses. As the beta-values of the left postcentral gyrus and right precuneus were highly correlated (*r* = 0.914) and the localization of these clusters was quasi-symmetrical across the hemispheres, they might reflect the bilateral activity of a functionally unified cluster. Thus, their activity was averaged into one predictor reflecting SMN activity.

**Table 2:** Regions showing increased error-related activation in OCD patients compared to healthy control participants.

**Peak Activity** **Neural Region**	**hem**	**x**	**y**	**z**	**zmax**	**k**

Supplementary Motor Area (SMA) extending to preSMA	R, L	2	−16	64	10.01	120
Precuneus	R	12	−46	62	9.94	165
Postcentral Gyrus	L	−24	−44	66	9.45	80

Note. Coordinates are refer to the standard template provided by the Montreal Neurological Institute (MNI). Hem = Hemisphere, R = right, L = left.

### 3.2 Sociodemographic and clinical data

During two pre-therapy diagnostic sessions, sociodemographic and clinical data were collected to assess eligibility. Lifetime diagnoses of mental disorders including personality disorders were assessed with the SCID-I and SCID-II, respectively (First et al., 1997, First et al., 1995). In order to quantify symptom severity, the following self-report questionnaires and clinical interviews for symptoms of obsessive–compulsive disorder and depression were applied before treatment, every 20th session and at termination of treatment: Y-BOCS, Obsessive-Compulsive Inventory-Revised (OCI-R, Foa et al., 2002), Beck-Depression-Inventory-II (BDI II, Beck, Steer, & Brown, 1996), Montgomery-Asperg Depression Rating Scale (MADRS, Montgomery & Asberg, 1979). For non-completers the Last Observation Carried Forward method was used as a conservative estimate for outcome data.

### 3.3 Data analysis

Separate hierarchical binary logistic regression analyses were applied for the prediction of response and remission. Response was defined according to the reliable change index (RCI, Jacobson, Follette, & Revenstorf, 1984). The RCI assesses whether an observed change in symptom score is statistically reliable, when considering the reliability and the standard deviation of the measure in the relevant population. The RCI was calculated for Y-BOCS total scores with an internal consistency of the Y-BOCS of α = 0.79 (Moritz et al., 2002) and a standard deviation of SD = 5.41. Using this metric, the critical difference for significant change of the Y-BOCS was 8 points. Remission was operationalized as a combination of post-treatment Y-BOCS score <= 12 (Mataix-Cols et al., 2016) and reliable symptom change.

As a priori correlation analyses revealed high collinearity between the brain activity of the SMA and the SMN (*r* > 0.76, *p* <.001), a separate regression analysis was conducted for each brain activity measure (SMA, SMN) and each outcome variable (response, remission), resulting in a total of four models. Separate analyses were conducted for the SMA and SMN clusters in order to allow for identification of their distinct contribution to treatment response prediction. To investigate whether error-related activity improves outcome prediction beyond the contribution of previously identified sociodemographic and clinical predictors (Kathmann et al., 2022, Keeley et al., 2008, Knopp et al., 2013), the following variables were included in the first step of the hierarchical regression: initial symptom severity (Y-BOCS), unemployment (categorical: currently employed vs currently unemployed), comorbid personality disorders (categorical: no comorbid diagnoses of personality disorder vs at least one comorbid personality disorder) and previous medication use (categorical: no previous psychoactive medication vs any previous or current psychoactive medication). Some previously identified predictors (family dysfunction, relationship status) could not be investigated, as they were not assessed in the present sample. Hoarding subtype was not investigated as patients with predominant hoarding symptoms were excluded from the fMRI study. In the second step, the respective brain measure (SMA activity, SMN activity) was entered into the model in order to investigate whether inclusion of error-related activity significantly improves treatment response prediction.

### 3.4 Exploratory and control analyses

In an additional exploratory-three-step model we investigated the effects of other clinically plausible predictor variables that were assessed in the present sample (comorbid axis I disorder, current medication use, initial severity of depressive symptoms). Here, previously established sociodemographic and clinical predictor variables were entered in the first step, exploratory clinical variables in the second step and brain activity measures in the third step. Additionally, a numbers of control analyses were conducted that assessed the effects of clinical and behavioral variables and possible interaction effects of the predictor. The specific models and their results are presented in the supplemental material. Taken together, the control analyses confirmed the stability and specificity of the main results reported below.

## 4 Results

### 4.1 Average symptom change

Dimensional symptom changes from pre- to post-treatment are presented in Table 3. The Y-BOCS score significantly decreased from pre-treatment to post-treatment, *t*(71) = 11.95, *p* <.001, with a mean reduction of 9.44 points and a large effect size of Cohen’s *d* = -1.31. The changes were significantly larger in completers than in non-completers, *t*(70) = 3.84, p <.001. The additional outcome measures also significantly improved from pre- to post-treatment (OCI-R: *t*(71) = 10.44, *p* <.001, MADRS: *t*(71) = 7.64, *p* <.001, BDI-II: *t*(71) = 8.61, *p* <.001). Here, changes were trend-level larger in completers than in non-completers (all *t* < 1.76, all *p* >.083 Completers and non-completers did not significantly differ in clinical symptoms at baseline (all *t* < -1.00, all p >.320).

**Table 3:** Pre- to post-therapy dimensional symptom change in primary (Y-BOCS) and secondary (OCI-R, MADRS, BDI-II) outcome measures in the whole sample (all), completers and non-completers.

		*M_pre_ (SD)*	*M_post_ (SD)*	*t*	*p*	*d*
all						
	Y-BOCS	22.01 (5.00)	12.57 (7.08)	11.95	<0.001	−1.77
	OCI-R	24.83 (11.96)	12.54 (10.61)	10.44	<0.001	−1.17
	MADRS	13.67 (9.58)	5.08 (5.84)	7.64	<0.001	−0.76
	BDI-II	18.71 (11.78)	8.23 (9.53)	8.61	<0.001	−0.94
Completers						
	Y-BOCS	21.72 (4.79)	10.91 (6.03)	13.48	<0.001	−2.03
	OCI-R	24.29 (11.55)	11.00 (9.43)	10.20	<0.001	−1.24
	MADRS	13.52 (9.85)	4.72 (6.02)	6.91	<0.001	−0.77
	BDI-II	18.03 (11.81)	6.77 (8.40)	8.97	<0.001	−1.06
Non-Completers						
	Y-BOCS	23.21 (5.82)	19.43 (7.19)	2.26	0.042	−0.69
	OCI-R	27.07 (13.79)	18.93 (13.0)	3.21	0.007	−0.84
	MADRS	14.29 (8.64)	6.57 (4.97)	3.16	0.008	−0.70
	BDI-II	21.50 (11.67)	14.29 (11.77)	2.09	0.057	−0.56

The categorical outcome measures presented in Table 4 illustrate that 55.6 % of patients showed a response and 38.9 % reached remission. Response, χ^2^ (1) = 11.99, *p* =.001, and remission rates, χ^2^ (1) = 7.37, *p* =.007, were significantly lower in non-completers than in completers.

**Table 4:** Categorical pre- to post-therapy symptom change (response, remission) in the whole sample (all), completers and non-completers.

	Response		Non-response		Remission		Non-Remission	
	*n*	%	*n*	%	*n*	%	*n*	%
All	40	55.6	32	44.4	28	38.9	44	61.1
Completer	38	65.5	20	34.5	27	46.6	31	53.4
Non-Completer	2	14.3	12	85.7	1	7.1	13	92.9

*Note.* Response was measured by the reliable change index. Remission was fulfilled for patients who showed reliable symptom change (response) and a post-therapy Y-BOCS score <= 12.

### 4.2 Prediction of reliable symptom change

The first block of the hierarchical logistic regression for response showed a classification accuracy of 63.9 %, and a trend-level model fit, Wald χ^2^ (4) = 8.23, *p* =.084, *R^2^* = 0.15. Higher initial symptom severity emerged as a significant predictor and was associated with a higher likelihood of response, *β* = 0.126, Wald χ^2^ (4) = 5.42, *p* =.020, OR = 1.13, 95 % CI [1.02, 1.26].

Inclusion of the error-related activity of the SMN in the second block resulted in a significant improvement of model fit, Wald χ^2^ (1) = 4.52, *p* =.033. The model showed a classification accuracy of 65.3 % and a significant model fit, Wald χ^2^ (5) = 12.75, *p* =.026, *R^2^* = 0.22. In addition to initial symptom severity, *β* = 0.133, Wald χ^2^ (1) = 5.61, *p* =.018, OR = 1.14, 95 % CI [1.02, 1.28], error-related SMN activity also was a trend-level predictor, *β* = 0.398, Wald χ^2^ (1) = 3.11, *p* =.078, OR = 1.49, 95 % CI [0.96, 2.32]. A higher error-related BOLD response of the SMN was associated with a higher likelihood of response.

Inclusion of the error-related activity of the SMA in the second block did not significantly increase model fit, Wald χ^2^ (1) = 2.31, *p* =.129.

### 4.3 Prediction of remission

The first block model did not reach a significant model-fit, Wald χ^2^ (4) = 4.92, *p* =.295, *R^2^* = 0.09. The second block models indicated that none of the brain activity significantly increased model fit (all Wald χ^2^ (1) < 0.80, all *p* >.372).

### 4.4 Discussion

The present study investigated the utility of increased error-related BOLD response of the SMA and SMN as potential biomarkers of treatment response in OCD. Error-related activity of the SMN contributed to the prediction of treatment response beyond the variance accounted for by previously established clinical and sociodemographic predictors. In line with previous findings, patients with stronger baseline abnormalities exhibited a higher probability for response. To the best of our knowledge, this is the first study to show that error-related SMN activity predicts CBT treatment response in OCD patients. This substantially extends the evidence for increased error-related brain activity in OCD (Norman et al., 2019, Riesel, 2019), by showing that the functional mechanism reflected in SMN activity alterations is related to treatment outcome. In contrast, the error-related activity of the SMA did not emerge as a significant treatment response predictor.

The SMN comprises regions involved in the integration of sensory information and the generation and control of motor behaviors (van den Heuvel et al., 2016). A substantial proportion of OCD patients (60–70 %) report that their compulsions are not only driven by fear/anxiety but also by sensory phenomena such as aversive or uncomfortable sensations or perceptions that could be related to dysfunctions of the SMN (Ferrão et al., 2012, Lee et al., 2009, Shavitt et al., 2014, Shephard et al., 2021). These sensory phenomena can manifest as “not-just-right”-experiences related to ordering, arranging, counting and repeating compulsions, but also as tactile sensations of feeling dirty in the context of cleaning/washing compulsions (Ferrão et al., 2012, Shephard et al., 2021). In line with this, larger gray matter volume (Subirà et al., 2015) and increased activation of the SMN (Brown et al., 2019) were observed in OCD patients with sensory phenomena. Thus, stronger error-related SMN activity might indicate that errors elicit aversive sensory phenomena in OCD patients which in turn create the urge to perform repetitive remedial actions. Furthermore, Shephard and colleagues (2021) proposed that increased SMN activity in OCD may also be related to the habit-like properties of compulsions in some OCD patients. In concert with subcortical structures the SMN is involved in habit formation (de Wit et al., 2012, Tricomi et al., 2009). Thus, increased SMN activity in OCD may contribute to the transition from goal-directed (i.e. performed to reduce fear/anxiety) to habitual compulsions (i.e. performed to reduce diffuse “not-just-right”-experiences) (Shephard et al., 2021).

Against this background, the predictive value of increased error-related SMN activity may be attributed to its capacity to adjust toward a more adaptive state through treatment. Olatunji et al. (2014) reported that increased activity of the emotion processing network and the SMN during symptom provocation predicted response to CBT in OCD patients with contamination compulsions. They concluded that this pattern reflects the activation of symptom-relevant fear networks, which according to the Emotional Processing Theory by Foa and Kozak (1986) is essential for reorganization of the fear-structure during exposure therapy. Similarly, increased activity of the SMN during error processing may reflect the activation of aversive sensory phenomena following errors (i.e. “not-just-right”-experiences). Thus, stronger task-related activation of the SMN may characterize patients that are less prone to engage in (cognitive) avoidance under symptom provocation and thus more likely to profit from exposure therapy (Foa and Kozak, 1986, Paul et al., 2016).

As there is consistent evidence for an increased ERN in patients with OCD (Riesel, 2019) and the ERN has been linked to activity of regions within the cingulo-opercular salience network, such as the midcingulate cortex and SMA (Debener et al., 2005, Grutzmann et al., 2016), we assumed that the error-related activity of the SMA might contribute to therapy response prediction. However, our analysis provided no evidence for a significant contribution of the SMA cluster beyond the variance accounted for by sociodemographic and clinical variables. There are several possible explanations for this distinction between the contribution of error-related SMA and SMN activity. Firstly, the error-related activity of the salience network and the SMN might reflect different aspects of cognitive-emotional error processing. While the activity of the salience network might be primarily related to initial error detection and categorization as a salient negative event, the activity of the SMN might be related to secondary emotional-sensory responses to errors. Secondly, EEG research indicates that the ERN reflects a trait-marker of psychopathology rather than a state-marker. Increased ERN amplitudes in OCD persist despite symptom reduction in CBT (Hajcak et al., 2008, Riesel et al., 2015), are also observed in unaffected first-degree relatives of OCD patients (Riesel et al., 2011, Riesel et al., 2019) and are thus discussed as a promising endophenotype candidate for OCD (Riesel, 2019). Against this background, the salience network activity reflected in the ERN might constitute a risk marker for disorder onset rather than a marker for its malleability by treatment. Thus, the utility of the ERN as predictor of treatment response in OCD should be investigated in future studies.

Additionally, it is notable that the SMN regions significantly contributing to the therapy response prediction are not located within the cingulo-opercular network, which constitutes the core error processing network in healthy populations and comprises the anterior and midcingulate cortices, the SMA and the anterior insulae (Norman et al., 2019, Taylor et al., 2007, Ullsperger et al., 2014). Although the flanker task successfully activated the cingulo-opercular salience network in OCD patients and healthy control participants, the group differences for the postcentral gyrus and precuneus are located outside these regions. In line with this, some previous studies have shown that error monitoring alterations in OCD might not only be characterized by an *increased activity* of the cingulo-opercular network, but also by broader activation within this network and the recruitment of additional brain regions (Fitzgerald et al., 2010, Grutzmann et al., 2016, Grützmann et al., 2021, Stern et al., 2011). In a combined EEG-FMRI study we observed that the increased ERN amplitude in OCD was related to an altered generator configuration: while the MCC contributed to ERN amplitude in both groups, an additional generator within the SMA was selectively present in the patient group (Grutzmann et al., 2016). A recent *meta*-analysis by Norman et al. (2019) confirmed increased error-related activation within the cingulo-opercular network in OCD, but also detected increased activation in the anterior lateral prefrontal cortex. The authors argue that these additional neural resources might reflect compensatory effort at engaging in behavioral corrections. In a similar vein, selective activation of the postcentral gyrus and precuneus in OCD may reflect the activation of additional processes in OCD that are not commonly involved in error-processing in healthy individuals, such as aversive sensory phenomena.

Some limitations of the current study have to be considered. The analysis tested the predictive value of error-related activity of a priori defined brain regions. Thus, it cannot be excluded that other brain regions may also constitute significant predictors. Additionally, although inclusion of the error-related SMN activity significantly increased model fit, the individual predictor contribution only reached a trend level. As this may be attributed to the relatively small sample, the predictive value of biomarkers should be further explored with larger samples. Furthermore, psychotherapy was not standardized and 46 % of the patients received additional psychoactive medication. This naturalistic approach is representative under routine care conditions in Germany but generalization to other treatment conditions as for example manualized and/or intensified CBT may be limited.

In conclusion, the present study significantly adds to the growing evidence that alterations of error-processing reflect an important mechanism in OCD pathology by showing that error-related SMN activity can serve as a predictor of CBT treatment response. Still, several question regarding clinical application need to be addressed. Firstly, brain activity measures are costly and time-intensive, which constitutes a disadvantage compared to routinely acquired clinical and sociodemographic data. Although the inclusion of error-related SMN activity significantly improved the prediction model in the current study, the absolute magnitude of the increase in classification accuracy was small. Thus, the clinical application of neuroimaging biomarkers of treatment response will have to be balanced against their cost-effectiveness (Hoexter et al., 2015). Regarding this criterion, resting state functional connectivity may be superior to task-related activity, as it can be assessed with shorter measurement sequences. Another promising approach may be to probe the brain-network dysfunctions and underlying pathogenic traits with other data sources that are easier to acquire such as EEG. Furthermore, although including brain activity measures significantly improves outcome prediction, classification accuracies still are below the threshold for actual clinical utility (Hilbert & Lueken, 2020). Additional and more sensitive features from brain activity patterns should be identified and combined in order to improve prediction accuracy. (Bowyer et al., 2019). Nevertheless, the current study illustrated that including neurobiological data has the potential to significantly improve model fit and classification accuracy.

## CRediT authorship contribution statement

**Rosa Grützmann:** Formal analysis, Investigation, Data curation, Writing – original draft, Visualization. **Julia Klawohn:** Conceptualization, Data curation, Writing – review & editing. **Björn Elsner:** Conceptualization, Data curation, Writing – review & editing. **Benedikt Reuter:** . **Christian Kaufmann:** Conceptualization, Data curation, Visualization. **Anja Riesel:** Conceptualization, Investigation. **Katharina Bey:** Conceptualization, Investigation. **Stephan Heinzel:** Conceptualization, Investigation. **Norbert Kathmann:** Funding acquisition, Project administration, Supervision.

## Declaration of Competing Interest

The authors declare that they have no known competing financial interests or personal relationships that could have appeared to influence the work reported in this paper.

## Data Availability

Data will be made available on request.

## Footnotes

1 These clusters were interpreted as indicators of DMN activity in our previous manuscript. A comparison with the seven-network brain parcellation mask by Thomas Yeo et al. (2011) in the current analysis indicated that although the clusters are spatially extent and comprise regions within the DMN, the peak voxels are located within the sensorimotor network.
