# Supplementary material for: Error-related activity of the sensorimotor network contributes to the prediction of response to cognitive-behavioral therapy in obsessive–compulsive disorder
Source: Neuroimage Clin. 2022 Sep 30;36:103216. doi: 10.1016/j.nicl.2022.103216 (PMC9668595; doi:10.1016/j.nicl.2022.103216)
Supplement: Supplementary data 1 [file mmc1.docx]

**Supplemental material 1: Exploratory analysis of additional clinical variables**

We conducted an additional exploratory analysis, in which we investigated whether other clinical variables assessed in the present study contribute to therapy response prediction beyond the variance accounted for by previously established socioeconomic and clinical predictors. To this end, a hierarchical logistic regression model with three steps was computed. In the first step, the following previously established predictors were included: initial symptom severity (Y-BOCS), unemployment (categorical: currently employed vs. currently unemployed), comorbid personality disorders (categorical: no comorbid diagnoses of personality disorder vs. at least one comorbid personality disorder) and previous medication use (categorical: no previous psychoactive medication vs. any previous or current psychoactive medication). In the second step, the following exploratory clinical predictors were included: comorbid axis I disorder (categorical: no comorbid diagnoses of axis I disorder vs. at least one comorbid axis I disorder), current medication use (categorical: no current psychoactive medication vs. any current psychoactive medication) and initial severity of depressive symptoms (MADRS). In the third step, the respective brain measure (SMA activity, SMN activity) was entered into the model in order to investigate whether inclusion of error-related activity significantly improves treatment response prediction.

The first block showed a classification accuracy of 63.9 %, and a trend-level model fit, Wald χ^2^ (4) = 8.23, *p* = .084, *R^2^* = 0.15. Higher initial symptom severity emerged as a significant predictor and was associated with a higher likelihood of response, *β* = .126, χ^2^ (1) = 5.42, *p* = .020, OR = 1.13, 95% CI [1.02, 1.26]. Inclusion of the exploratory clinical predictors in the second block did not significantly improve model fit, Wald χ^2^ (3) = 4.22, *p* = .238. Inclusion of the error-related activity of the SMN in the third block resulted in a significant improvement of model fit, Wald χ^2^ (1) = 4.15, *p* = .042. The model showed a classification accuracy of 70.8 % and a significant model fit, Wald χ^2^ (8) = 16.60, *p* = .035, *R^2^* = 0.28. In addition to initial symptom severity, *β* = .134, Wald χ^2^ (1) = 3.53, *p* = .060, OR = 1.14, 95% CI [0.99, 1.31], previous medication use, *β* = 1.13, Wald χ^2^ (1) = 3.37, *p* = .066, OR = 3.08, 95% CI [0.93, 10.24], current medication use, *β* = -.981, Wald χ^2^ (1) = 2.86, *p* = .091, OR = 0.38, 95% CI [0.12, 1.17], and error-related SMN activity also emerged as trend-level predictors, *β* = .447, Wald χ^2^ (1) = 2.91, *p* = .088, OR = 1.56, 95% CI [0.93, 2.62]. A higher error-related BOLD response of the SMN was associated with a higher likelihood of response. Interestingly, previous and current medication use were associated with likelihood of therapy response in different directions: While previous medication use was associated with a higher likelihood of therapy response, current medication use was associated with a lower likelihood of therapy response.

Inclusion of the error-related activity of the SMA in the third block resulted in a trend-level improvement of model fit, Wald χ^2^ (1) = 2.98, *p* = .085. The model showed a classification accuracy of 75 % and a trend-level model fit, Wald χ^2^ (8) = 15.43, *p* = .051, *R^2^* = 0.26. In addition to initial symptom severity, *β* = .124, Wald χ^2^ (1) = 3.34, *p* = .068, OR = 1.13, 95% CI [0.99, 1.29], previous medication use, *β* = 1.27, Wald χ^2^ (1) = 4.18, *p* = .041, OR = 3.56, 95% CI [1.05, 12.05] and current medication use, *β* = -1.01, Wald χ^2^ (1) = 2.90, *p* = .088, OR = 0.37, 95% CI [0.12, 1.16] also emerged as trend-level predictors. Even though the inclusion of the error-related SMA activity led to an overall increase in model fit, the individual contribution of this predictor did not reach significance, *β* = .326, Wald χ^2^ (1) = 2.62, *p* = .106, OR = 1.39, 95% CI [0.93, 2.06].

These models again confirm, that the inclusion of error-related SMN activity can significantly improve therapy response prediction in OCD beyond the contribution of previously established clinical and sociodemographic predictors and exploratory clinical predictors. It is notable that, in contrast to previous medication use, current medication use was associated with a lower likelihood for therapy response in the current sample. Importantly, this effect cannot be attributed to differences in symptom severity (i.e. patients with higher symptom having a higher likelihood of medication use), as it was observed while controlling for initial Y-BOCS scores. One possible explanation might be related to the different treatment rationales for medication and psychotherapy. Medication treatment is based on a biological model of disorder development and comprises a passive role for the patient. Opposed to this, CBT is based on a complex disorder model with an emphasis on the role of dysfunctional cognitions and behavioral patterns in symptom maintenance. Thus, CBT requires the patients to actively address their symptoms, which can be a demanding, effortful and stressful process. Symptoms reductions in CBT can lead to secondary effects such as increases in locus of control and self-efficacy that strengthen the patient’s belief in the therapy rationale and thereby their compliance. However, patients who receive psychoactive medication in addition to CBT may primarily attribute these effects to the medication, thereby strengthening the biological disease and treatment model and reducing this cascade of positive psychotherapeutic effects.

**Supplemental material 2: Control analyses assessing additional clinical characteristics**

***S2.1 Therapy response prediction in the completers sample***

In order to assess, whether the effects of the main analysis are also present in a subsample of patients who completed the psychotherapy treatment, we applied the binary hierarchical logistic regression in the completers sample.

The model fit of the first block of the hierarchical logistic regression for response did not reach significance, Wald χ^2^ (4) = 7.70, *p* = .103, *R^2^* = 0.17. Inclusion of the error-related activity of the SMN in the second block resulted in a trend-level improvement of model fit, Wald χ^2^ (1) = 3.16, *p* = .075. The model showed a classification accuracy of 72.4 % and a trend-level model fit, Wald χ^2^ (5) = 10.86, *p* = .054, *R^2^* = 0.24. Initial symptom severity emerged a significant predictor, *β* = .181 Wald χ^2^ (1) = 6.36, *p* = .012, OR = 1.12, 95% CI [1.04, 1.38], while the individual contribution of error-related SMN activity did not reach significance, *β* = .374, Wald χ^2^ (1) = 2.11, *p* = .146, OR = 1.45, 95% CI [0.87, 2.41]. Higher initial symptom severity was associated with a higher likelihood of response.

Inclusion of the error-related activity of the SMA in the second block did not result in a significant improvement of model fit, Wald χ^2^ (1) = 2.22, *p* = .136.

The logistic regression analysis for remission in the completers sample replicated the previous non-significant results: The first block model did not reach a significant model-fit, Wald χ^2^ (4) = 4.979, *p* = .310, *R^2^* = 0.11. The second block models indicated that none of the brain activity significantly increased model fit (all Wald χ^2^ (1) < .64, all *p* > .425).

Taken together, the general pattern of results in the completers sample was comparable to the intention-to-treat sample. Inclusion of the error-related SMN activity in the logistic regression analysis for response led to a trend-level increase in model fit. Additionally, the general direction of the effect was confirmed, i.e. higher error-related SMN activity was associated with a higher likelihood of response, albeit its individual contribution did not reach statistical significance. Two factors may contribute to the reduced statistical significance: 1) reduced sample size (*n* = 58 vs. *n* = 72) resulting in reduced statistical power and 2) significantly lower number of non-responders in the completers sample resulting in reduced variance of the dependent variable.

***S2.2 Predictive value of the number of therapy sessions***

As patients differed in the number of therapy session they had received, we conducted a control analysis in which we assessed the predictive value of the number of therapy sessions for therapy response prediction. To this end, the step-wise logistic regression comprised three steps: 1) inclusion of previously established sociodemographic and clinical predictors, 2) inclusion of number of therapy sessions and 3) inclusion of error-related SMN or SMA activity (separate models). Results for the first block were identical to those reported in the manuscript. Inclusion of the number of therapy sessions in the second block did not significantly improve model fit, Wald χ^2^ (1) = 0.02, *p* = .901. Inclusion of the error-related activity of the SMN in the third block resulted in a significant improvement of model fit, Wald χ^2^ (1) = 4.58, *p* = .032. The model showed a classification accuracy of 68.1 % and a significant model fit, Wald χ^2^ (6) = 12.82, *p* = .046, *R^2^* = 0.22. In addition to initial symptom severity, *β* = .134, Wald χ^2^ (1) = 5.64, *p* = .018, OR = 1.14, 95% CI [1.02, 1.28], error-related SMN activity also was a trend-level predictor, *β* = .401, Wald χ^2^ (1) = 3.15, *p* = .076, OR = 1.49, 95% CI [0.96, 2.33]. A higher error-related BOLD response of the SMN was associated with a higher likelihood of response.

Inclusion of the error-related activity of the SMA in the third block did not result in a significant improvement of model fit, Wald χ^2^ (1) = 2.31, *p* = .128.

***S2.3 Predictive value of depressive symptoms***

We conducted a control analysis in which we assessed the predictive value of initial depressive symptoms for therapy response prediction. The control analysis comprised three steps: 1) inclusion of previously established sociodemographic and clinical predictors, 2) inclusion of initial severity of depressive symptoms (MADRS) and 3) inclusion of error-related SMN or SMA activity (separate models).

Results for the first block were identical to those reported in the manuscript. Inclusion of initial depressive symptoms (MADRS) in the second block did not significantly improve model fit, Wald χ^2^ (1) = 0.04, *p* = .949. Inclusion of the error-related activity of the SMN in the third block resulted in a significant improvement of model fit, Wald χ^2^ (1) = 4.52, *p* = .033. The model showed a classification accuracy of 65.3 % and a significant model fit, Wald χ^2^ (6) = 12.75, *p* = .047, *R^2^* = 0.22. In addition to initial symptom severity, *β* = .135, Wald χ^2^ (1) = 4.36, *p* = .037, OR = 1.14, 95% CI [1.01, 1.30], error-related SMN activity also was a trend-level predictor, *β* = .399, Wald χ^2^ (1) = 3.09, *p* = .079, OR = 1.49, 95% CI [0.96, 2.33]. A higher error-related BOLD response of the SMN was associated with a higher likelihood of response.

Inclusion of the error-related activity of the SMA in the third block did not result in a significant improvement of model fit, Wald χ^2^ (1) = 2.32, *p* = .127.

**Supplemental material 3: Control analysis assessing behavioral parameters**

As FMRI measures are costly and difficult to collect, it is important to assess whether behavioral parameters might probe the same functional process and thereby yield similar predictive value. The control analysis comprised three steps: 1) inclusion of previously established sociodemographic and clinical predictors, 2) inclusion of error rate and 3) inclusion of error-related SMN or SMA activity (separate models).

Results for the first block were identical to those reported in the manuscript. Inclusion of error rate in the second block did not significantly improve model fit, Wald χ^2^ (1) < 0.01, *p* = .990. Inclusion of the error-related activity of the SMN in the third block resulted in a significant improvement of model fit, Wald χ^2^ (1) = 4.60, *p* = .032. The model showed a classification accuracy of 66.70 % and a significant model fit, Wald χ^2^ (6) = 12.82, *p* = .046, *R^2^* = 0.22. In addition to initial symptom severity, *β* = .135, Wald χ^2^ (1) = 5.67, *p* = .017, OR = 1.15, 95% CI [1.02, 1.28], error-related SMN activity also was a trend-level predictor, *β* = .404, Wald χ^2^ (1) = 3.15, *p* = .076, OR = 1.50, 95% CI [0.96, 2.34]. A higher error-related BOLD response of the SMN was associated with a higher likelihood of response.

Inclusion of the error-related activity of the SMA in the third block did not result in a significant improvement of model fit, Wald χ^2^ (1) = 2.32, *p* = .128.

**Supplemental material 4: Control analysis assessing interaction effects**

***S4.1 Interactions between clinical/sociodemographic variables and error-related brain activity***

To explore possible interactions between clinical/sociodemographic variables and error-related brain activity in the therapy response prediction, we added the interaction terms between the block one and block two variables in the third block of the hierarchic logistic regression. Predictor variables in the third block were: initial symptom severity (Y-BOCS) * brain activity (SMN, SMA), comorbid personality disorder * brain activity (SMN, SMA), unemployment * brain activity (SMN, SMA), previous medication use * brain activity (SMN, SMA). Results for the first and second block were identical to those presented in the manuscript. Including the third block did not result in a significant improvement of therapy response prediction, neither for the SMN model, Wald χ^2^ (4) = 1.86, *p* = .762, nor for the SMA model, Wald χ^2^ (4) = 4.00, *p* = .407.

***S4.1 Interactions between the number of therapy sessions and clinical/sociodemographic variables and error-related brain activity***

To explore possible interactions between the number of therapy sessions and clinical/sociodemographic variables as well as error-related brain activity in therapy response prediction, we added the interaction terms between number of therapy sessions and the block one (previously established socio-demographic and clinical variables) and block two variables (error-related SMN/ SMA activity, separate models) in the third step of the hierarchical logistic regression. Predictor variables in the third block were: number of therapy sessions, number of therapy session * initial symptom severity (Y-BOCS), number of therapy session * comorbid personality disorder, number of therapy session * unemployment, number of therapy sessions *previous medication, number of therapy sessions * error-related SMN activity. Results for the first and second block were identical to those presented in the manuscript. Including the third block did not result in a significant improvement of therapy response prediction, neither for the SMN model, Wald χ^2^ (6) = 7.80, *p* = .253, nor for the SMA model, Wald χ^2^ (6) = 10.26, *p* = .114.
